# Case Report: Prenatal presentation of Masson's tumor: first reported case and review of the literature

**DOI:** 10.3389/fped.2026.1636879

**Published:** 2026-01-22

**Authors:** Bahattin Tanrıkulu, Ayça Erşen Danyeli, M. Memet Özek

**Affiliations:** 1 Department of Neurosurgery, Division of Pediatric Neurosurgery, Acibadem University School of Medicine, Istanbul, Türkiye; 2 Department of Pathology, Division of Neuropathology, Acibadem University School of Medicine, Istanbul, Türkiye

**Keywords:** intravascular papillary endothelial hyperplasia, Massons' s tumor, prenatal, thalamic tumor, neonatal brain

## Abstract

Intravascular papillary endothelial hyperplasia (IPEH), or Masson's tumor, is a rare, benign vascular lesion that can closely resemble malignant vascular tumors. While primarily diagnosed in adulthood, pediatric cases are uncommon, and no prenatal diagnoses have been reported to date. Here, we present the first documented prenatal presentation of fetal intracranial IPEH detected *in utero* at 34 weeks of gestation. This case highlights the importance of considering IPEH in the differential diagnosis of fetal intracranial masses and underscores the role of early prenatal detection in optimizing perinatal and surgical management.

## Introduction

Intravascular papillary endothelial hyperplasia (IPEH), or Masson's tumor, is a rare benign vascular lesion, with intracranial cases accounting for only a small proportion of all reported cases (1). While primarily observed in adults, pediatric cases have also been documented. Due to its histopathological resemblance to malignant vascular tumors, intracranial IPEH presents a significant diagnostic challenge and may be misclassified as a more aggressive lesion.

Here, we present the first reported prenatal presentation of intracranial IPEH; the definitive diagnosis was established postnatally by histopathology. This case highlights the implications for early detection, management, and prognosis.

## Case presentation

A 34-week-old fetus was diagnosed with an intracranial mass lesion during routine prenatal ultrasonographic follow-up. An intrauterine MRI subsequently revealed a left thalamic tumor, leading the mother to visit our clinic for further consultation. We recommended a comprehensive evaluation with contrast-enhanced cranial MRI during the neonatal period.

The baby was delivered by cesarean section at 36 weeks of gestation, weighing 2,600 g at birth. A contrast-enhanced cranial MRI performed at 3 days of age demonstrated a highly vascularized mass lesion with significant contrast uptake, causing midline shift, resulting in hydrocephalus and inducing extensive edema in the surrounding parenchyma, including the left temporal lobe. A preoperative computerized tomography (CT) scan further confirmed the presence of excessive blood content within the tumor.

Surgical intervention was performed on postnatal day 7 via a left parietal transsulcal approach. Intraoperatively, the lesion appeared dark red, was extremely firm, and had a well-defined cleavage plane separating it from the surrounding thalamic tissue. The procedure was uneventful. Although significant intraoperative bleeding had been anticipated, blood loss was less than expected.

**Figure 1:**
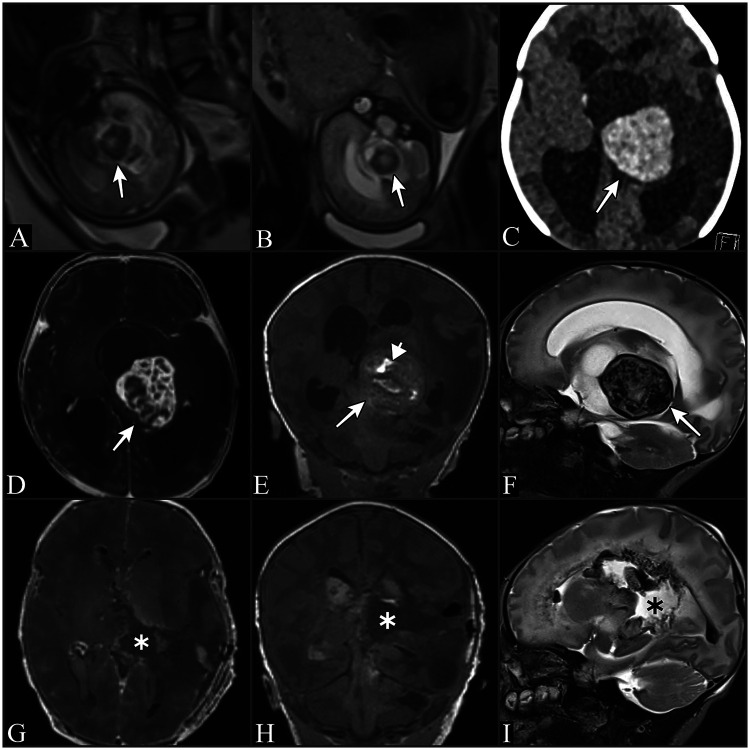
**(A,B)** fetal MRI of baby showing T2 hypointense mass lesion located in the midline structures of the brain (arrows). **(C)** Axial section cranial CT scan showing hyperdense mass lesion within left thalamus (arrow). **(D)** Axial section T1 weighted cranial MRI scan with contrast shows mass lesion with excess contrast uptake within left thalamus (arrow). **(E)** Coronal section T1 weighted MRI without contrast shows lesion (arrow) with hyperintense areas highlighting blood accumulation within the lesion (arrowhead). **(F)** The lesion was hypointense in T2 weighted images. **(G)** Axial section T1 weighted contrasted cranial MRI, **(H)** Coronal section T1 weighted cranial MRI without contrast, **(I)** Sagittal section T2 weighted image. **(G,H,I)** images shows gross total resection of the tumor (asterisks).

Postoperatively, the infant was monitored in the neonatal intensive care unit on room air and exhibited no neurological deficits. On postoperative day 1, partial tonic-clonic seizures occurred but were fully controlled with phenobarbital. An early postoperative MRI confirmed complete resection of the lesion (Figure 1). She was discharged on postoperative day 7 in stable condition.

**Figure 2:**
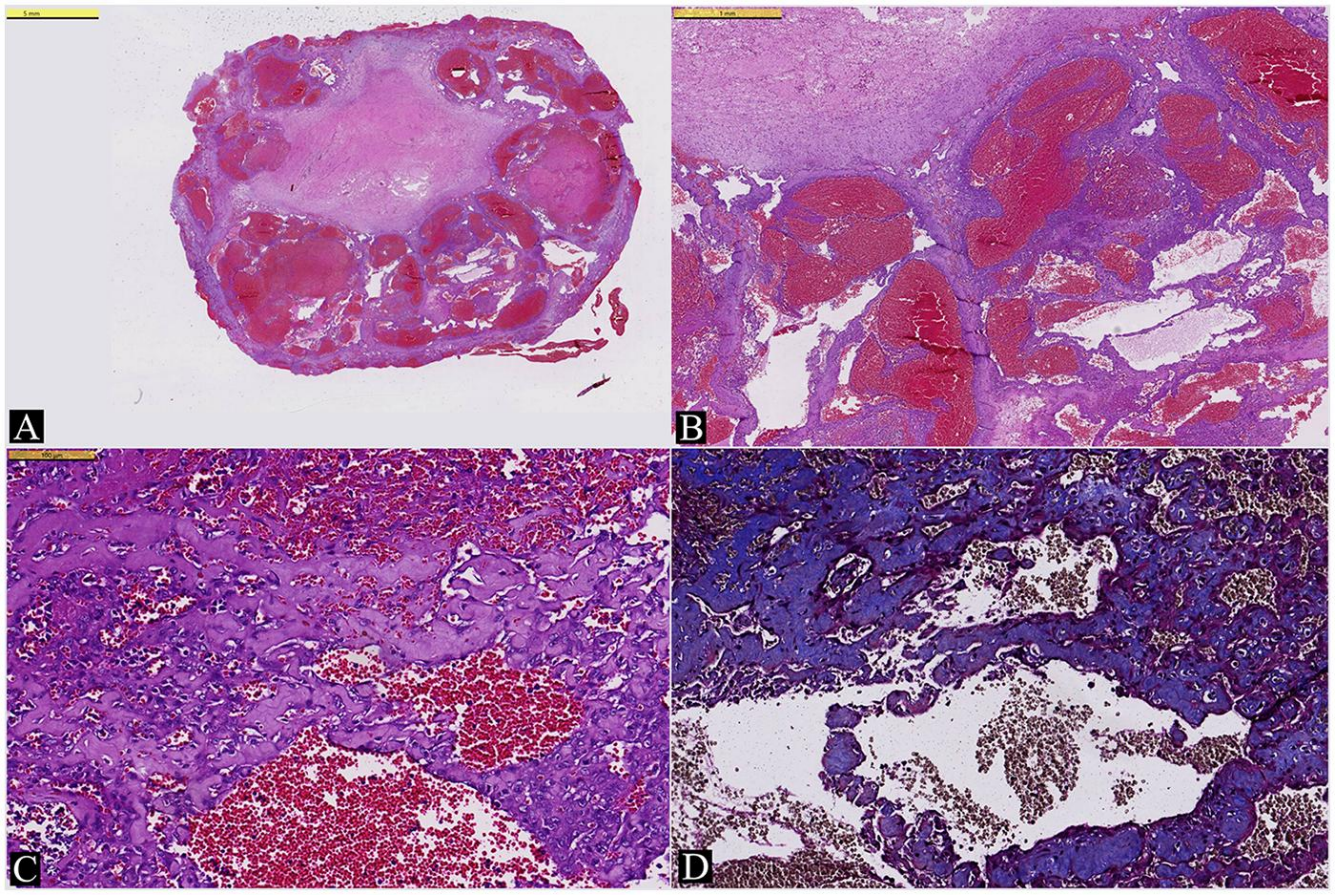
**(A)** The lesion is well-circumscribed and composed of cavernous vascular structures filled with blood (HE stain, 0.64× magnified). **(B)** Endothelial proliferation is present within the lumina of these cavernously dilated vascular structures, featuring papillary growth and anastomosing formations. (HE stain, 3× magnified). **(C)** At high magnification, reactive endothelial cells appear atypical and may mimic malignant cells. (HE stain, 21× magnified). **(D)** Masson's trichrome stain highlights the hyalinized stroma of the papillary structures (Masson's trichrome stain, 17×).

For histopathologic evaluation, the entire lesion was processed. On microscopic examination, it was well-demarcated from the surrounding tissue and composed of dilated vascular structures resembling cavernous vessels. Within these vascular structures, proliferation of endothelial cells with swollen cytoplasm was observed, giving rise to numerous papillary formations, some exhibiting anastomosing patterns. The stroma of these papillary structures and anastomosing cord-like formations was hyalinized, as demonstrated by Masson's trichrome stain. The pathology of the lesion was reported as “Intravascular Papillary Endothelial Hyperplasia (Masson's Tumor)” (Figure 2).

Acıbadem Brain Tumor next-generation sequencing (NGS) panel and tumor methylation analysis (Heidelberg Brain Tumor Classification version 12.8) were performed to rule out any neoplastic process. No pathogenic molecular alterations were detected. The copy number variation profile was flat, and the methylation pattern did not match any known tumor methylation class.

## Discussion

Masson's tumor, also known as intravascular papillary endothelial hyperplasia (IPEH), is a rare benign vascular lesion characterized by reactive endothelial cell proliferation (2). First described by Masson in 1923, IPEH can mimic aggressive vascular neoplasms such as angiosarcoma due to its histopathological features, including endothelial cell proliferation, papillary structures within a thrombotic background, and immunopositivity for endothelial markers such as CD31, CD34, and factor VIII (1). While it commonly affects the extremities, lips, orbits, and salivary glands, intracranial involvement is exceedingly rare (3). In the congenital brain-tumor differential, both highly aggressive and benign entities may appear similar on prenatal or early neonatal imaging—including atypical teratoid rhabdoid tumor (ATRT), malignant teratomas, and astrocytomas (4). Because imaging appearances can overlap and prove misleading—especially when IPEH is considered—obtaining appropriate diagnostic tissue is key to accurate classification and management (5). Including the present case, there have been 11 pediatric intracranial IPEH cases reported in the literature so far (1, 6) (Table 1).

**Table 1:** Literature review of pediatric papillary endothelial hyperplasia (Masson tumor) cases (5, 6).

Authors and year	Age/Sex	Clinical presentation	Location	Treatment Course	Outcome
Nagib et al., 1982	16 yrs, F	Seizures	Multiple intracranial supratentorial lesions	Subtotal resection	Reoperation 19 mo later, no recuurence in 9 yrs
Chen et al., 1984	3.5 mo, F	Seizures, increased ICP	Frontal lobe	Biopsy	Died 6 mo later
Sickler et al., 1990	12 day, F	Increased ICP	Temporal lobe	Total resection	N/A
Wen et al., 1991	15 day, F	Increased ICP	Confluens sinum	Subtotal	Recur 2 mo later, treated by Chx, stabilized
De Plessis et al., 2003	6 yrs, F	Skull bump	Parietal and frontal lobes	Total resection	No recurrence at 1 year
Cagli et al., 2004	16 yrs, F	Unilateral CN III-IV deficit	Cavernous sinus	Subtotal	No regrowth of residual lesion within 3 yrs.
Shih et al., 2012	2 day, M	Proptosis	Supracellar, orbital, cerebellar	Subtotal	Resected in 9 mo of age, died 6 mo later.
Park et al., 2012	10 yrs, F	Skull bump	Frontal lobe	Total resection	No recurrence at 8 mo
Shah et al., 2014	3 mo, M	Skull bump	Parietal lobe	Total resection	No recurrence at 1 yr
Mann et al., 2016	4 yrs, F	N/A	Parietal lobe	N/A	N/A
Current case	34 weeks of gestation, M	During Prenatal USG	Thalamus	Total resection	Resected in 7 days of age, no recurrence at 3 mo follow-up

Chx, cehmotherapy, CN, cranial nerve, ICP, intracranial pressure, mo, month, N/A, not available, USG, ultrasonography, yr, year.

When present within the central nervous system, IPEH can arise in association with pre-existing vascular malformations, aneurysms, thrombi, angioma, lymphangioma leading to a spectrum of clinical manifestations dictated by its location (7). Given its rarity in the intracranial compartment, preoperative diagnosis is often challenging, necessitating careful histopathological and immunohistochemical evaluation to distinguish it from malignant vascular tumors (8).

On CT scans, IPEH typically appears hyperdense, reflecting its highly vascular nature and blood-engorged structure. On MRI, these tumors generally present as iso- to hypointense on T1-weighted images and iso- to hyperintense on T2-weighted images. In some cases, they may appear hyperintense on T1 and hypointense on T2, indicating the presence of blood within the lesion. T1-weighted MRI with contrast usually demonstrates homogeneous contrast enhancement, highlighting the tumor's hypervascularity (3, 8).

The present case also appeared hyperdense on CT scans. On MRI, it was iso- to hyperintense on T1-weighted images and hypointense on T2-weighted images due to the increased blood content. Additionally, it exhibited marked contrast enhancement, indicating its highly vascular nature.

Although rare, IPEH should be considered in the differential diagnosis of intracranial vascular lesions. A key distinguishing feature of IPEH, differentiating it from other intracranial vascular lesions such as angiosarcoma, cavernoma, and angiomatous meningioma, is its consistent confinement within the intravascular lumen (8, 9).

For an accurate histopathological assessment, total excision of the lesion and demonstration of clear demarcation from the surrounding tissue are crucial. At high magnification, marked cytological atypia and frequent mitotic figures may be observed, potentially mimicking a malignant tumor. Therefore, low magnification examination is important to appreciate the well-circumscribed nature of the lesion and recognize the characteristic endothelial proliferation with anastomosing and papillary structures.

Histologically, intravascular papillary endothelial hyperplasia (IPEH) is recognized in three forms: a pure (primary) intravascular form arising within a normal—typically venous—channel; a mixed (secondary) form occurring in association with a pre-existing vascular anomaly such as an arteriovenous malformation (AVM), hemangioma, or varix; and a rare extravascular form that develops within an organized hematoma. Recognizing these subtypes helps explain the diverse clinical contexts in which IPEH presents (10–12).

IPEH is considered a benign tumor, and complete resection is generally curative. Sim et al. reported a case of parasellar IPEH along with a literature review, identifying 22 previously reported adult and pediatric cases of intracranial IPEH. They noted that among 10 patients who underwent gross total resection, none experienced recurrence. However, in 5 out of 12 patients (42%) who underwent subtotal resection, recurrence was observed during follow-up (13).

Due to the high risk of recurrence following partial resection, some authors recommend more aggressive adjuvant treatments, such as stereotactic or Gamma Knife radiosurgery and chemotherapy (8). However, others have suggested a possible link between radiation therapy and the development of IPEH due to radiation-induced endothelial hyperplasia (8, 14). Nevertheless, in cases where complete resection poses greater risks than benefits, radiation-based therapies may be a viable option, provided that patients are closely monitored during follow-up (8).

The present case represents the first reported fetal IPEH in the literature.

We suggest that in pediatric patients, given the importance of complete resection for prognosis and the potential adverse effects of radiotherapy in partially resected cases, total tumor removal should be prioritized whenever feasible. Neonatal IPEH has been summarized by Shih and colleagues, who reported a neonatal intracranial case and reviewed two prior neonatal presentations. Across these reports, hemorrhage often dominated the clinical picture; importantly, subtotal/partial resection was associated with serious hemorrhagic complications and poor outcomes. These observations reinforce the value of gross-total excision when safely achievable (2). On the other hand, the developing brain's neuroplasticity offers a greater capacity for functional recovery, making aggressive resection a more favorable option. In contrast, in adult patients with tumors in eloquent locations, where complete removal may lead to catastrophic neurological deficits, a more conservative approach with partial resection may be preferable, accompanied by close follow-up and consideration of adjuvant treatment options as needed.

## Conclusion

This case represents the first reported fetal IPEH in the literature, highlighting the rarity of intracranial involvement in this benign vascular lesion. Given its potential to mimic malignant vascular tumors, accurate diagnosis requires careful histopathological and immunohistochemical evaluation. Complete tumor removal should be the preferred approach whenever feasible. In cases where only partial resection is possible, adjuvant treatment options and close follow-up should be considered due to the risk of recurrence.

## Data Availability

The raw data supporting the conclusions of this article will be made available by the authors, without undue reservation.
